# Diagnostic value of magnetic resonance imaging and multi-slice spiral computed tomography in the diagnosis of pancreatitis

**DOI:** 10.12669/pjms.41.11.12753

**Published:** 2025-11

**Authors:** Xiaojuan Tian, Kaiyi Liang, Lingxia Jiang, Xin Guo

**Affiliations:** 1 Xiaojuan Tian, Department of Radiology, Jiading District Central Hospital, Affiliated Shanghai University of Medicine & Health Sciences, Shanghai 201800, P.R. China; 2 Kaiyi Liang, Department of Radiology, Jiading District Central Hospital, Affiliated Shanghai University of Medicine & Health Sciences, Shanghai 201800, P.R. China; 3 Lingxia Jiang, Department of Radiology, Jiading District Central Hospital, Affiliated Shanghai University of Medicine & Health Sciences, Shanghai 201800, P.R. China; 4 Xin Guo, Department of Radiology, Jiading District Central Hospital, Affiliated Shanghai University of Medicine & Health Sciences, Shanghai 201800, P.R. China

**Keywords:** Diagnostic, Pancreatitis, Magnetic resonance imaging, Multi-slice spiral computed tomography

## Abstract

**Objective:**

This study aimed to compare the diagnostic value of magnetic resonance imaging (MRI) and multi-slice spiral computed tomography (MSCT) in patients with pancreatitis.

**Methodology:**

Clinical data of 128 patients with suspected pancreatitis who underwent MRI and MSCT examinations in Jiading District Central Hospital from November 2022 to September 2024 were retrospectively analyzed. Of them, 64 patients were eventually diagnosed with pancreatitis and 64 received a different diagnosis. Pancreatitis detection rate, diagnostic sensitivity, specificity, accuracy, positive predictive value (PPV) and negative predictive value (NPV) of MRI and MSCT, as well as the performance of both methods in identifying focal or total pancreatic enlargement and peripancreatic exudate, were compared.

**Results:**

The sensitivity (96.88%), accuracy (96.09%) and NPV (96.83%) of MRI in diagnosing pancreatitis were significantly better than those of MSCT (84.38%, 89.06%, 85.71%, respectively, P<0.05). Both methods had comparable specificity (MRI: 95.31%, MSCT: 93.75%) and PPV (MRI: 95.38%, MSCT: 93.10%) (P>0.05). The detection rate of MRI in identifying pancreatic enlargement (96.00%) and peripancreatic exudate (94.23%) was superior to that of MSCT (82.00% and 76.92%, respectively, P<0.05).

**Conclusions:**

Both MRI and MSCT are high-value diagnostic tools for pancreatitis. However, MRI performs better in identifying pancreatic parenchymal changes and surrounding tissue involvement. Based on the results of this study, clinicians should prioritize MRI as an imaging evaluation method for pancreatitis when possible.

## INTRODUCTION

Pancreatitis is often manifested as severe upper abdominal pain, with or without nausea, vomiting and elevated serum amylase levels.^1^,^2^ If not identified and intervened promptly, patients may rapidly develop severe acute pancreatitis that can lead to a life-threatening multiple organ dysfunction syndrome.^1^-^3^ Due to the lack of specific clinical manifestations in the early stages of the disease, accurate diagnosis of pancreatitis is still challenging.^3^,^4^ Therefore, finding sensitive, specific and reproducible imaging diagnostic tools has become one of the current research priorities.^2^-^4^

Imaging examinations play a central role in the diagnosis and assessment of pancreatitis by detecting changes in pancreatic structure, necrosis, exudation and morphology of the adjacent tissues.^5^ Multi-slice spiral computed tomography (MSCT) is widely used in the preliminary assessment of pancreatitis in clinical practice due to its fast scanning speed and high spatial resolution.^5^,^6^ However, while MSCT can clearly display the contour, density changes and peripheral vascular involvement of the pancreas, there are certain limitations in identifying early small soft tissue changes.^5^-^7^ Magnetic resonance imaging (MRI) has higher soft tissue contrast, especially in displaying pancreatic parenchyma and its surrounding exudate and necrosis,^8^,^9^ with feasible functional evaluation and no need for radiation. However, the examination time for an MRI is relatively long and requires high patient cooperation, as well as specialized equipment.^9^

Given the differences in imaging mechanisms and application characteristics between MRI and MSCT, the clinical comparison of their diagnostic efficacy remains of practical significance. This retrospective study aimed to compare the detection rates and diagnostic efficacies of MRI and MSCT techniques in the diagnosis of pancreatitis, providing evidence-based support for optimizing the selection of clinical examination methods and improving the early diagnosis rate of this disease.

## METHODOLOGY

This single-center, retrospective, controlled study included 128 patients suspected of having pancreatitis who underwent imaging examinations at Jiading District Central Hospital from November 2022 to September 2024. According to the final clinical pathological diagnosis, 64 patients diagnosed with pancreatitis were included in the pancreatitis group. An additional 64 patients with imaging and clinical manifestations suggesting other acute abdominal conditions and ultimately excluding pancreatitis were selected as the non-pancreatitis group. The non-pancreatitis group included diseases such as cholecystitis, common bile duct stones, acute gastroenteritis, gastric ulcer perforation, appendicitis and non-specific abdominal pain to improve comparability between groups and avoid diagnostic interference. All patients underwent dual imaging examinations using MRI and MSCT upon admission.

### Ethical Approval:

The ethics committee of our hospital approved this study with the number LL20250004, Date: June 2025.

### Inclusion Criteria:

- Patients meet at least two of the following diagnostic criteria for acute pancreatitis based on the Revised Atlanta Classification (2012).^10^
- Typical clinical manifestations (e.g., persistent upper abdominal pain).
- Serum amylase and/or lipase levels ≥ three times the upper limit of normal.
- Characteristic imaging findings of acute pancreatitis.
- Complete clinical data.
- Good imaging quality.

### Exclusion criteria:

- Patients with moderate to severe renal dysfunction and inability to tolerate contrast agents.
- Other malignant or benign tumors.
- Quality of the images impacted by motion artifacts, which prevent proper diagnostics.
- Patients with concomitant bleeding disorders or coagulation dysfunction.

### MRI

The China United Imaging uMR 780 3.0T superconducting magnetic resonance system was used. The patient was scanned in a supine position, using a 12-channel body coil with a layer thickness of 6 mm and a layer spacing of 0.6 mm. The scanning sequence included:

- T1 weighted image (QUICK3D FS transverse section, TE=1.56 ms, TR=3.41 ms).
- T2 weighted image (FSE FS TRIG transverse position, TE=88.88 ms, TR=4505 ms).
- T2 weighted coronal image (SSFSE sequence, TE=97.28 ms, TR=1100 ms).
- Three-dimensional MR cholangiopancreatography (3D MRCP sequence, TE=650 ms, TR=3350 ms).

### MSCT

A combined uCT960 and 320 slice spiral CT scanner was used. The patient was placed in a supine position. The scanning parameters were set as follows: tube current 220 mA, tube voltage 120 kV, layer thickness 2 mm and pitch 3:1. Patients were instructed to take 500 ml of warm water orally 30 minutes before the examination for gastrointestinal filling. The preliminary plain scan covered the top of the liver to the lower pole of the kidney, followed by injection of iobitridol (manufacturer: Guerbet, France; Specification 100 ml) through the elbow vein at a rate of 3 ml/s. Arterial phase (delayed by 25 s), portal phase (delayed by 50 s) and equilibrium phase (delayed by 120 s) enhanced scans were sequentially performed. All images were reconstructed by the workstation and used for image analysis. Two senior professional radiologists independently reviewed all images, analyzed them blindly and recorded the results. If necessary, a consensus was reached through negotiation.

### Statistical analysis:

All data were analyzed using SPSS 25.0 software (IBM Corp, Armonk, NY, USA). Measurement data were expressed as mean and standard deviation (SD) and an independent sample t-test was used for intergroup comparison. Count data were expressed as n (%) and the chi-square test was used to test for intergroup differences. P<0.05 indicated a statistically significant difference. The detection rates of MRI and MSCT in patients with pancreatitis were compared. Sensitivity, specificity, positive predictive value (PPV) and negative predictive value (NPV) were calculated to determine the diagnostic value of MRI and MSCT for pancreatitis. The ability of MRI and MSCT to identify focal/total pancreatic enlargement and peripancreatic exudate were compared. Diagnostic performance indicators, including sensitivity, specificity, positive predictive value (PPV), and negative predictive value (NPV), were calculated using the following standard formulas: Sensitivity = TP / (TP + FN), Specificity = TN / (TN + FP), PPV = TP / (TP + FP), and NPV = TN / (TN + FN), where TP, FP, FN, and TN represent true positive, false positive, false negative, and true negative cases, respectively.

## RESULTS

This study included 128 patients suspected of having pancreatitis. Among them, there were 56 males and 72 females; the age range of the cohort was 20-74 years, with an average age of 46.3 ± 12.2 years. The final clinical and pathological diagnosis confirmed pancreatitis in 64 cases (Table-I). There was no statistically significant difference (P>0.05) in age, gender, body mass index, symptom distribution, interval between onset and examination, smoking and alcohol consumption history between the two groups of patients. Table-I.

**Table-I:** Comparison of general information.

Information	Pancreatitis (n=64)	Non-pancreatitis (n=64)	t/χ^2^	P
Age (years)	47.4±11.6	45.2±12.8	1.011	0.314
Female (yes), n (%)	37 (57.81)	35 (54.69)	0.127	0.722
Body Mass Index (kg/m^2^)	24.2±3.2	23.4±2.9	1.495	0.137
Interval from onset to examination (hours)	41.9±10.8	44.4±10.9	-1.271	0.206
The main symptoms, n (%)			1.300	0.729
Vomited	14 (21.9)	16 (25.0)		
Nauseated	19 (29.7)	15 (23.4)		
Abdominal pain	25 (39.1)	29 (45.3)		
Jaundice	6 (9.4)	4 (6.3)		
Smoking history (yes), n (%)	28 (43.75)	25 (39.06)	0.290	0.590
Drinking history (yes), n (%)	35 (54.69)	31 (48.44)	0.501	0.479

As shown in Table-II, MRI detected pancreatitis in 62 out of 64 pancreatitis patients, with a sensitivity of 96.88%. Among patients in the non-pancreatitis group, MRI excluded pancreatitis in 61 cases, with a specificity of 95.31%. MSCT was able to confirm the diagnosis in 54 patients (84.38%) with pancreatitis and excluded pancreatitis in 60 out of 64 patients in the non-pancreatitis group with a specificity of 93.75%.

**Table-II:** Analysis of detection of pancreatitis.

Diagnostic method	n	True Positive (TP)	False Positive (FP)	False Negative (FN)	True Negative (TN)
MRI	128	62	3	2	61
MSCT	128	54	4	10	60

***Note:*** TP = patients correctly identified as having pancreatitis; FP = patients incorrectly identified as having pancreatitis; FN = patients with pancreatitis missed by imaging; TN = patients correctly identified as not having pancreatitis. “Positive” refers to imaging results indicating pancreatitis; “Negative” refers to imaging results not indicating pancreatitis.

As shown in Table-III, the sensitivity of MRI in diagnosing pancreatitis was 96.88% (62/64), the accuracy was 96.09% (123/128) and the NPV was 96.83% (61/63), all significantly higher than MSCT (84.38%, 89.06%, 85.71%, respectively, all P<0.05). There was no statistically significant difference between the two in terms of specificity (MRI: 95.31%, MSCT: 93.75%, P=1.000) and PPV (MRI: 95.38%, MSCT: 93.10%, P=0.586).

**Table-III:** Comparison of diagnostic efficacy for pancreatitis.

Diagnostic methods	Sensitivity	Specificity	Accuracy	negative predictive value	positive predictive value
MRI	96.88% (62/64)	95.31% (61/64)	96.09% (123/128)	96.83% (61/63)	95.38% (62/65)
MSCT	84.38% (54/64)	93.75% (60/64)	89.06% (114/128)	85.71% (60/70)	93.10% (54/58)
*χ^2^*	5.885	0.000	4.605	4.987	0.297
*P*	0.015	1.000	0.032	0.026	0.586

***Note:*** Sensitivity refers to the proportion of true pancreatitis cases correctly identified by the imaging modality; specificity refers to the proportion of non-pancreatitis cases correctly excluded; accuracy indicates the overall proportion of correctly classified cases; PPV (positive predictive value) is the probability that a positive imaging result truly indicates pancreatitis; NPV (negative predictive value) is the probability that a negative imaging result truly excludes pancreatitis. Sensitivity and NPV were considered of particular clinical relevance in this study, as they reflect the modality’s reliability in minimizing missed diagnoses, especially in early-stage or mild pancreatitis.

**Fig.1:**
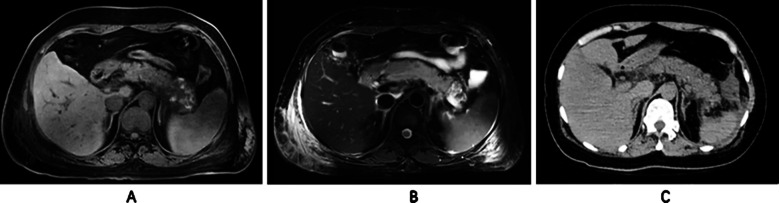
Female, 40 years old, diagnosed with pancreatitis. *A*. T1WI showing an increase in pancreatic volume and significant peripancreatic exudation. High signal intensity is observed in the pancreatic area on T1WI, indicating local bleeding. *B*. T2WI showing uneven signal intensity in the pancreatic parenchyma, suggesting necrotic changes. *C*. MSCT imaging, showing an enlarged pancreatic contour with blurred edges and patchy low-density shadows around the pancreas, supporting exudation.

MRI detected 48/50 cases (96.00%) of focal or overall enlargement of the pancreas, which was significantly higher than MSCT [41/64 cases (82.00%) (*χ^2^*=5.005, P=0.025)]. Table-IV. MRI was able to detect 49/52 cases (94.23%) of peripancreatic exudate, which was also superior to the detection rate of MSCT [40/52 cases (76.92%) (*χ^2^*=6.310, P=0.012)]. A significant difference in the ability of both techniques to detect local pancreatic lesions and surrounding exudate is illustrated in Fig.1. These two imaging features-pancreatic enlargement and peripancreatic exudation-are widely recognized as important indicators of disease activity and severity in acute pancreatitis. Therefore, their accurate identification contributes not only to early diagnosis but also to clinical staging and prognosis evaluation.

**Table-IV:** Comparison of detection of pancreatic partial/total enlargement and peripancreatic exudate.

Diagnostic methods	partial / total enlargemen (n=50)	peripancreatic (n=52)
MRI	48 (96.00)	49 (94.23)
MSCT	41 (82.00)	40 (76.92)
*χ^2^*	5.005	6.310
*P*	0.025	0.012

## DISCUSSION

This study demonstrated that MRI had significantly better sensitivity (96.88%), accuracy (96.09%) and negative predictive value (96.83%) than MSCT (P < 0.05) in the overall detection of pancreatitis. This study suggests that MRI has a higher recognition rate in identifying pancreatitis, especially in mild or early cases, which may be attributed to its excellent soft tissue resolution, allowing for the clear detection of subtle inflammatory changes, such as those in the pancreatic parenchyma, peripancreatic fat space and focal exudate.^8^-^14^ These results are consistent with the previous studies. A meta-analysis by Ha et al.^10^ found that MRI is significantly more sensitive and accurate than CT in distinguishing autoimmune pancreatitis from pancreatic cancer.

A multicenter study of pediatric chronic pancreatitis by Trout et al.^12^ also confirmed that MRI has a higher observer consistency in the assessment of pancreatic parenchymal changes. Ilhan et al.^13^ found that the diffusion-weighted MRI was superior to enhanced CT in identifying mild edema, early necrosis lesions and pancreatic function assessment of pancreatic parenchyma in acute biliary pancreatitis. The sensitivity and negative predictive value results of this study further confirm these observations. In the early stage of pancreatitis, the lesions are often characterized by mild histological changes, such as infiltration of inflammatory cells and early edema.^15^ MRI has high sensitivity to such early soft tissue changes and can clearly display subtle changes within the pancreatic parenchyma.^13^,^14^ In contrast, MSCT is prone to overlooking some early mild lesions due to its relative limitations in soft tissue comparison.^8^,^16^

In terms of the recognition capabilities of two technologies in key pathological features, this study demonstrated that the detection rate of MRI in detecting focal or overall enlargement of the pancreas (96.00% vs 82.00%) and peripancreatic exudate (94.23% vs 76.92%) was significantly better than that of MSCT (P<0.05). Pancreatic enlargement and peripancreatic exudate are important imaging markers of disease activity and severity. The high detection rate of MRI, thus, provides more comprehensive support for clinical disease grading and the formulation of personalized treatment strategies.^17^-^19^ In agreement with these results, Xiao et al.^17^ showed that the multi-parameter MRI has unique advantages in evaluating the distribution of pancreatic and surrounding tissue lesions, which helps to determine the presence of necrotic or liquefied lesions and guide puncture or drainage treatment.

Notably, this study did not detect a statistically significant difference between MRI and MSCT in terms of specificity and PPV (P>0.05). This suggests that MSCT still has high diagnostic efficacy in identifying typical cases of pancreatitis and excluding non-pancreatitis cases. MSCT offers the advantages of fast imaging speed and convenient operation, enabling the quick determination of lesion extent and screening for complications in critically ill patients in emergency settings. Therefore, it remains an important method for imaging screening of clinical pancreatitis.^5^,^6^,^20^,^21^ A study by Grassedonio et al.^20^ showed that MSCT performs well in identifying local complications such as fluid accumulation, pseudocysts and necrotic lesions in acute pancreatitis and is particularly suitable for early decision-making to determine whether surgical or interventional treatment is needed. From a safety perspective, MRI does not use ionizing radiation, making it particularly suitable for patients with chronic pancreatitis, young adults and patients with renal insufficiency who require repeated follow-up evaluations.^17^-^19^,^22^ On the other hand, MSCT poses radiation risks and is dependent on contrast agents, requiring a comprehensive evaluation based on the patient’s baseline condition.^20^,^21^

This study contributes novel evidence to the existing literature by directly comparing MRI and MSCT in the same patient cohort using a unified diagnostic standard. The findings confirm that MRI demonstrates superior sensitivity, negative predictive value, and accuracy in detecting pancreatitis, particularly in early or mild cases. This has important clinical implications for individualized imaging strategies, suggesting that MRI may be prioritized for patients requiring refined disease grading, long-term follow-up, or radiation avoidance, whereas MSCT remains a valuable option for rapid assessment in emergency settings. Key strengths of this study include the use of a standardized diagnostic framework (Revised Atlanta Classification), dual imaging examinations in all subjects to eliminate inter-patient variability, and blinded image evaluation by experienced radiologists to enhance objectivity. Nonetheless, limitations such as the single-center, retrospective design and the absence of classification-based subgroup analysis warrant caution. Future research should adopt multicenter, prospective approaches and explore diagnostic performance across different types of pancreatitis, including necrotizing, edematous, and chronic forms, while assessing their correlation with clinical outcomes.

### Limitations

Although this study provides comparative evidence between MRI and MSCT in the diagnosis of pancreatitis, it has several limitations. Firstly, this is a single-center retrospective study with a limited sample size, which may result in selection bias. Secondly, subgroup analysis was not conducted on the classification of pancreatitis (such as mild, edematous, necrotic), resulting in a lack of more detailed stratification information. Thirdly, this study did not conduct follow-up verification on the correlation between imaging parameters and the clinical prognosis of patients. Future research can combine a prospective design and long-term follow-ups to explore the role of MRI and MSCT in the monitoring and prognostication of pancreatitis.

## CONCLUSION

Both MRI and MSCT are advantageous in the diagnosis of pancreatitis. MRI is associated with a higher sensitivity and image quality in identifying substantive lesions, assessing the extent of inflammation and grading the condition, making it suitable for refined evaluation and efficacy tracking of chronic cases. In contrast, MSCT is more timely and convenient in the acute stage of the disease and may be preferred for preliminary screening and the identification of severe complications. The decision to use MRI or MSCT should be based on the severity of the patient’s condition, examination objectives and individual differences, optimizing the diagnostic process and enhancing the support of imaging for the management of pancreatitis.

### Authors’ contributions:

**XT:** Literature search, study design and manuscript writing.

**KL, LJ and XG:** Data collection, data analysis and interpretation. Critical Review.

**XT:** Manuscript revision and validation and is responsible for the integrity of the study.

All authors have read and approved the final manuscript.
